# Dietary supply with polyunsaturated fatty acids and resulting maternal effects influence host – parasite interactions

**DOI:** 10.1186/1472-6785-13-41

**Published:** 2013-10-31

**Authors:** Nina Schlotz, Dieter Ebert, Dominik Martin-Creuzburg

**Affiliations:** 1 Limnological Institute, University of Konstanz, Konstanz 78464, Germany; 2 Zoological Institute, University of Basel, Basel 4051, Switzerland

**Keywords:** Arachidonic acid, *Daphnia magna*, Eicosapentaenoic acid, Food quality, Host parasite interactions, Immunity, Nutrition, *Pasteuria ramosa*, Resistance

## Abstract

**Background:**

Interactions between hosts and parasites can be substantially modulated by host nutrition. Polyunsaturated fatty acids (PUFAs) are essential dietary nutrients; they are indispensable as structural components of cell membranes and as precursors for eicosanoids, signalling molecules which act on reproduction and immunity. Here, we explored the potential of dietary PUFAs to affect the course of parasitic infections using a well-established invertebrate host – parasite system, the freshwater herbivore *Daphnia magna* and its bacterial parasite *Pasteuria ramosa.*

**Results:**

Using natural food sources differing in their PUFA composition and by experimentally modifying the availability of dietary arachidonic acid (ARA) and eicosapentaenoic acid (EPA) we examined PUFA-mediated effects resulting from direct consumption as well as maternal effects on offspring of treated mothers. We found that both host and parasite were affected by food quality. Feeding on C20 PUFA-containing food sources resulted in higher offspring production of hosts and these effects were conveyed to a great extent to the next generation. While feeding on a diet containing high PUFA concentrations significantly reduced the likelihood of becoming infected, the infection success in the next generation increased whenever the maternal diet contained PUFAs. We suggest that this opposing effect was caused by a trade-off between reproduction and immunity in the second generation.

**Conclusions:**

Considering the direct and maternal effects of dietary PUFAs on host and parasite we propose that host – parasite interactions and thus disease dynamics under natural conditions are subject to the availability of dietary PUFAs.

## Background

Resistance of animals to parasitic infections is influenced by various factors, among them genetic predisposition, environmental conditions, and nutritional state [1]. The role of nutrition in infectious diseases has been extensively investigated, as it is thought to affect establishment, pathogenesis, and duration of infections (e.g. [2-4]). The consensus is that under- or malnutrition impairs immunocompetence leading to increased susceptibility to and severity of infection. However, it becomes increasingly clear that disease patterns generated by the diet can be much more complex. Host – parasite interactions can be affected by the foraging activity per se [5-7], the amount of available food, as well as its quality [8,9]. While the search for food often establishes the contact between host and pathogen, food quantity and quality may play a role later in the infection process. Infected hosts and their parasites compete for the same nutrients acquired by the host [10]; i.e. nutrient supply could have direct effects on growth and reproduction of the host and simultaneously on the performance of the parasite. Moreover, certain components of the host’s defence mechanisms could be affected by dietary nutrients and, in consequence, indirectly influence pathogen success [11]. In contrast to what is often seen in mammals, food quantity limitation of the invertebrate host seems to impair the parasite, resulting in reduced within-host proliferation and decreased transmission [12-16].

Although still in their early stage, the combined efforts of nutritional ecology and eco-immunological research have brought to light exciting aspects of food quality effects under parasite challenge in invertebrates. For example, ratios of dietary protein to carbohydrates or dietary carbon (C) to phosphorus (P) have been shown to modify the incidence and intensity of infections [17-19]. While dietary deficiencies in elements can have severe consequences for the consumer’s fitness [20] there are other essential nutrients which have rarely been considered in research on the role of nutrient supply in pathophysiology of invertebrate hosts.

A dietary deficiency in polyunsaturated fatty acids (PUFAs) can severely constrain growth and reproduction of consumers [21-23]. Under parasite challenge, PUFA requirements may change and single PUFAs may be assigned to other roles. Three of the C20 PUFAs – arachidonic acid (ARA, 20:4n-6), eicosapentaenoic acid (EPA, 20:5n-3), and dihomo-γ-linolenic acid (DGLA, 20:3n-6) – are the substrates for a family of hormone-like substances called eicosanoids, which in vertebrates and invertebrates act on reproduction, the immune system, and ion transport physiology [24]. The importance of an adequate functioning of the arachidonic acid cascade for host defence mechanisms has been demonstrated in experiments in which animals were unable to clear an imposed bacterial infection when eicosanoid biosynthesis was blocked; this block could be bypassed by the injection of ARA into the body cavity [25].

In order to shed light upon the potential of dietary PUFAs to modulate infection in invertebrates we used the freshwater crustacean *Daphnia magna*, which is well understood regarding its nutritional ecology. An adequate dietary supply with PUFAs has been shown to support proper growth and reproduction and to influence temperature acclimation [26-29]. Furthermore, first evidence suggests that eicosanoids are active in *Daphnia* physiology [30,31] and that the eicosanoid biosynthesis machinery responds to the level of dietary precursor PUFAs [32]. To challenge our host, we chose *Pasteuria ramosa*, a castrating endoparasitic bacterium, for combined life history – infection experiments. The *D. magna* – *P. ramosa* system has been thoroughly investigated [33] and several aspects of the infection process and the inheritance of resistance have been elucidated [34,35].

Depending on the conditions experienced by mothers, eggs may be provisioned differentially with nutrients. Thus, offspring performance can greatly be affected by stress- or resource-related maternal effects [36-42]. *Daphnia* preferentially allocates PUFAs into their eggs [43]. Hence, if dietary PUFAs have the potential to influence an infection when consumed directly, offspring of mothers differing in their dietary PUFA provisioning might experience the same benefit or harm even if they do not have access to dietary C20 PUFAs.

Here, we provided hosts (*D. magna*) with food sources differing in their PUFA content and composition and additionally manipulated a diet deficient in C20 PUFAs by ARA and EPA supplementation. Subsequently, we reared offspring of mothers raised on the different food regimes exclusively on the C20 PUFA-deficient food to be able to assess PUFA-related maternal effects. Animals of both generations were exposed to the parasite (*P. ramosa*) and fitness consequences were recorded as host reproductive success, susceptibility to the parasite and within-host reproduction of the parasite.

## Results

### Elemental and biochemical composition of the food sources

The algal food organisms were characterized by low molar carbon to nitrogen (C:N) and carbon to phosphorus (C:P) ratios, i.e. high contents of nitrogen and phosphorus (Table 1). As the C:P ratios of the algae were rather low, a P-limitation of the host could be excluded. Moreover, C:P ratios within the range observed here (~100-230) are unlikely to change the elemental conditions within the host in a way that the parasite’s establishment or growth is hampered [18].

**Table 1:** **Elemental nutrient ratios (molar) and PUFA content (μg mg C**^**-1**^**) of the three food organisms**

	***S. obliquus***	***N. limnetica***	***Cryptomonas *** **sp.**
C:N	13.7 ± 0.0	13.0 ± 0.6	5.4 ± 0.0
C:P	232.9 ± 4.6	162.2 ± 3.9	100.1 ± 3.2
18:2n-6 (LIN)	45.5 ± 1.6	8.5 ± 0.4	10.2 ± 0.2
18:3n-3 (ALA)	62.4 ± 4.0	*n.d*	50.9 ± 1.1
18:4n-3 (STA)	8.5 ± 0.3	*n.d*	17.9 ± 0.4
20:3n-6 (DGLA)	*n.d*	2.2 ± 0.4	*n.d*
20:4n-6 (ARA)	*n.d*	24.5 ± 1.1	*n.d*
20:5n-3 (EPA)	*n.d.*	121.6 ± 1.1	45.5 ± 1.0
22:6n-3 (DHA)	*n.d.*	*n.d*	4.6 ± 0.0

Data are means of three replicates ± s.d. (*n.d*. = not detectable). Food suspensions consisting of *S. obliquus* and PUFA -containing liposomes contained either 26.1 ± 0.4 ARA or 20.3 ± 0.7 EPA (all values in μg mg C^-1^ ± s.d.), respectively.

Fatty acid profiles differed considerably between the three algae, especially with regard to PUFAs (Table 1). *S. obliquus* contained linoleic acid (LIN, 18:2n-6), high amounts of α-linolenic acid (ALA, 18:3n-3), and stearidonic acid (STA, 18:4n-3), but no PUFAs with more than 18 C atoms. In contrast, the PUFA composition of *N. limnetica* was characterized by the presence of DGLA and ARA as well as exceptionally high amounts of EPA. C18 PUFAs were present only in very low concentrations or were not detectable at all in *N. limnetica*. *Cryptomonas* sp. contained the three C18 PUFAs LIN, ALA, and STA and, additionally, considerable amounts of EPA, albeit in much lower concentrations than *N. limnetica*, and small amounts of DHA.

### PUFA profiles of *D. magna* eggs

**Figure 1:**
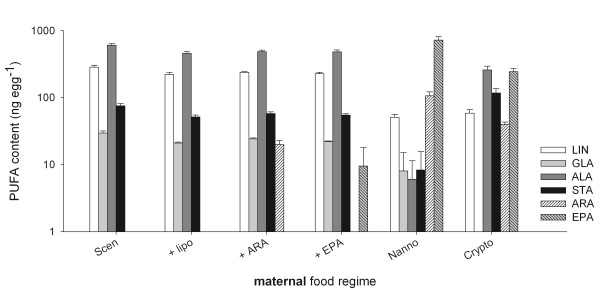
**PUFA content of second clutch eggs (ng egg**^**-1**^**).** Eggs collected from mothers raised on *S. obliquus* (Scen), *S. obliquus* supplemented with either control liposomes (+ lipo) or liposomes containing ARA or EPA (+ARA, + EPA), *N. limnetica* (Nanno), or *Cryptomonas* sp. (Crypto). Data are presented on a logarithmic scale as means of three replicates ± s.d.

Eggs basically reflected the PUFA composition of their mothers’ food source. In eggs produced on a *S. obliquus* diet no PUFAs of more than 18 C atoms could be detected (Figure 1). Eggs of *N. limnetica*-consuming mothers contained considerable amounts of ARA and EPA. When mothers where raised on *Cryptomonas* sp., their eggs contained EPA and also low amounts of ARA, although ARA could not be detected in *Cryptomonas* sp. Supplementation of *S. obliquus* with control liposomes did not affect the PUFA composition of the produced eggs. In contrast, low amounts of ARA or EPA were detected in eggs produced on ARA- or EPA-supplemented *S. obliquus*, indicating that these supplemented PUFAs were allocated into the eggs (Figure 1).

### Susceptibility of the host

**Figure 2:**
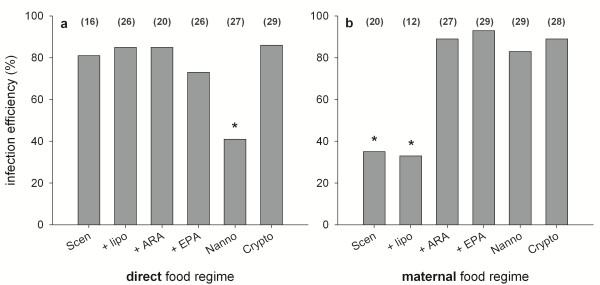
**Infection efficiency of *P. ramosa *in *D. magna*. a)** Animals raised on different food sources directly. **b)** Animals raised exclusively on *S. obliquus*, but mothers raised on different food sources. Data indicate the percentages of infected animals after exposure to the parasite (total numbers of individuals are given in brackets). Asterisks indicate a significant deviation from the grand mean (general linear hypothesis testing following GLM).

The parasite’s success in establishing an infection in spore-exposed hosts varied with food quality, regardless of whether the food sources were consumed directly (factor “food”, df = 5, deviance = 16.58, *p* < 0.01; Figure 2a) or were experienced only as maternal provisioning in the second generation experiment, where all offspring were raised on *S. obliquus*, irrespective of the food regimes their mother were raised on (factor “food”, df = 5, deviance = 37.65, *p* < 0.001; Figure 2b). However, direct and maternal effects differed substantially in pattern and extent. When animals were raised directly on the different food sources, the infection efficiency dropped significantly on a *N. limnetica* diet. Only ~40% of exposed animals were infected, which is a 6-fold decrease (odds ratio) compared to the *S. obliquus* diet (~80%). The other food treatments did not induce significant changes in infection efficiency (Figure 2a). The second generation experiment revealed that the maternal food regime strongly influenced the infection success of the parasite. Although all offspring fed exclusively on *S. obliquus*, the proportion of infected animals increased ~ 6-fold (odds ratio; from ~35% to >80%) when mothers were raised on diets containing C20 PUFAs, i.e. *N. limnetica*, *Cryptomonas* sp., as well as ARA- and EPA-supplemented *S. obliquus*.

### Reproductive success of healthy and infected hosts

**Figure 3:**
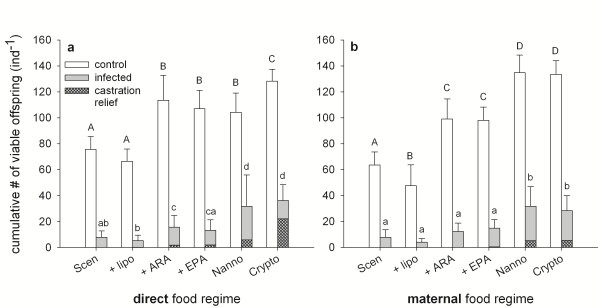
**Cumulative numbers of viable offspring produced by uninfected and *P. ramosa*-infected *D. magna*. a)** Animals raised on different food sources directly. **b)** Animals raised exclusively on *S. obliquus*, but mothers raised on different food sources. Shaded areas indicate the proportion of total offspring produced after the sterile phase (castration relief). Error bars indicate s.d. Bars labelled with the same letters are not significantly different (general linear hypothesis testing, p < 0.05 following GLM).

The cumulative numbers of viable offspring produced by healthy and *P. ramosa*-infected *D. magna* during the experiments were influenced by the quality of the different food source, both when these food sources were consumed directly (Figure 3a, Table 2) and when they were used as maternal food sources only (Figure 3b, Table 2). Strikingly, direct and maternal effects generated very similar patterns. When directly consumed, long-chain PUFAs increased offspring production of control (i.e. non-exposed) animals up to the level obtained with *N. limnetica* as food. Animals feeding on *Cryptomonas* sp. produced the highest numbers of offspring. These effects were conveyed to the next generation. In the maternal effects experiment, control animals whose mothers were provided with ARA or EPA produced significantly more offspring than those from mothers without dietary ARA or EPA supply. This trans-generational food quality effect was even stronger when *N. limnetica* or *Cryptomonas* sp. were used as maternal food source.

**Table 2:** Results of statistical analysis of the cumulative number of offspring using a generalized linear model

	**Cumulative number of host offspring**				
**(1)***direct*	df	deviance	residual df	residual deviance	*p*
*subset control* “food”	5	494.38	98	181.37	< 0.001
*subset infected* “food”	5	1035.1	128	812.2	< 0.001
**(2)***maternal*					
*subset control* “food”	5	685.94	76	131.35	< 0.001
*subset infected* “food”	5	481.41	104	482.33	< 0.001

Error distribution = quasi-Poisson, link function = log. (1) *D. magna* raised under different food regimes (direct supply). (2) *D. magna* raised under the same food regime (*S. obliquus*), but mothers raised under different food regimes (maternal effects).

*P. ramosa* is a castrating parasite and thus greatly impacts the fitness of its host. In accordance with what was seen in earlier studies [44], parasite-induced mortality was absent during the experimental period. However, infected animals of all treatments showed a distinct decrease in the production of viable offspring (Figure 3). Total numbers of offspring produced by infected animals were comparable between both direct (Figure 3a) and maternal (Figure 3b) food regimes. Supplementation of *S. obliquus* with ARA or EPA significantly increased offspring production of infected animals relative to the liposome control treatment in the mother generation (directly feeding on the different food sources), but this trend was not significant in infected animals of the next generation. In both generations, offspring numbers produced by infected animals were significantly higher when *N. limnetica* and *Cryptomonas* sp. were provided as food source. When feeding on PUFA-rich diets directly, infected hosts were able to produce offspring after the sterile phase caused by *P. ramosa* (Figure 3, hatched areas). This ‘castration relief’ was most prominent on a *Cryptomonas* sp. diet where more than 50% of total offspring were produced after the sterile phase. This restart of reproduction could be observed also, albeit to a lower extent, on *N. limnetica* as well as ARA- and EPA-supplemented *S. obliquus*. In the second generation experiment, animals started to reproduce again only when their mothers were raised on either *N. limnetica* or *Cryptomonas* sp.

### Spore production by the parasite

**Figure 4:**
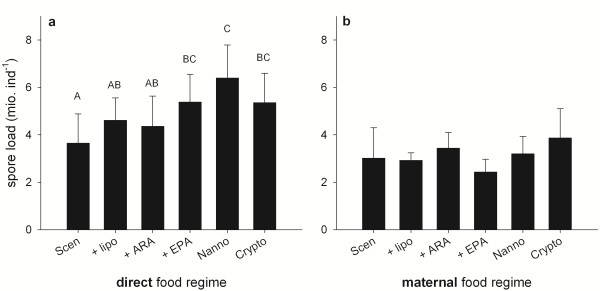
**Number of endospores counted in *P. ramosa*-infected *D. magna *27 days post infection. a)** Animals raised on different food sources directly. **b)** Animals raised exclusively on *S. obliquus*, but mothers raised on different food sources. Data are means of n = 10 ± s.d. Bars labelled with the same letters are not significantly different (Tukey’s HSD test, p < 0.05 following ANOVA). Treatments in b) did not differ statistically.

The life cycle of *P. ramosa* within its host ends with the formation of endospores in the body cavity and thus the spore load can be used as a proxy for the reproductive success of the parasite [33]. In the first generation experiment, when exposed directly to the different food regimes, the total number of endospores per individual host was affected by food quality (factor “food”; per individual: F_5, 54_ = 6.18, *p* < 0.001; per mg dry mass: df = 5, F = 4.67, p < 0.01; Figure 4a). The spore load per individual was significantly higher in animals raised on, *N. limnetica*, *Cryptomonas* sp., or EPA-supplemented *S. obliquus* as compared to animals raised on unsupplemented *S. obliquus*. Compared to the liposome control treatment, however, only animals raised on *N. limnetica* had significantly higher spore loads (Tukey’s HSD, p < 0.05). In the second generation experiment, food quality mediated effects on the total number of endospores per individual were virtually absent (factor “food”, F_5, 54_ = 0.95, p = 0.49; Figure 4b).

## Discussion

The potential of dietary PUFAs to modulate vertebrate and invertebrate physiology has intrigued researchers for decades. However, their role in host – parasite interactions and the consequent ecological significance are yet to be revealed. By providing our invertebrate host with food sources differing in their PUFA content and composition, we investigated direct and maternal effects of dietary PUFAs on the outcome of an infection with a bacterial parasite. Our results show that dietary PUFAs influence host – parasite interactions both when provided with the diet and when derived from maternal resources.

### PUFA-mediated food quality affects the susceptibility to and the severity of infection

By using a compatible host – parasite pair we could attribute the observed food quality effects specifically to changes in the ability of the host to cope with the parasite after it entered the host [34]. Whether the host can initially prevent the establishment of the parasite must therefore be associated with differences in within-host defence mechanisms. Feeding on *N. limnetica*, which contains high concentrations of ARA and EPA, resulted in a 6-fold reduction of the host’s susceptibility to infection. As the clearance of the invading parasite is an event very early in the infection process it is rather unlikely that competition for resources plays a role (assuming it does, a high quality food like *N. limnetica* would lead to higher infection rates). Thus, the biochemical composition of *N. limnetica*, characterized by high ARA and EPA concentrations, is most likely responsible for the higher resistance to infection. ARA and EPA serve as precursors for eicosanoids, signalling molecules which are known to modulate invertebrate immune responses [24]. It has been shown that feeding on diets differing in their PUFA composition can affect the expression of key enzymes within the eicosanoid pathway in *Daphnia*[32], suggesting that the high dietary supply with PUFAs has supported a more pronounced immune response to the invading parasite resulting in increased resistance. Transcriptomic and metabolomic studies will help to elucidate links between defence mechanisms and the eicosanoid pathway in *Daphnia*. Supplementation of *S. obliquus* with ARA or EPA did not lead to higher resistance, possibly because a higher concentration or the combination of both PUFAs is required to obtain a similar effect as observed with *N. limnetica*.

An alternative explanation for the increased resistance against *P. ramosa* on a *N. limnetica* diet could be directly related to effects of the exceptionally high amounts of EPA present in this alga. Although PUFAs are typically covalently bound to lipids in living tissue, free PUFAs might be released from phospholipids of *N. limnetica* as a consequence of cell damage during the feeding process of *D. magna*[45]. Free PUFAs are cytotoxic and bactericidal [46] and thus may have directly impaired the invading bacterium. However, we did not find evidence for the release of free PUFAs out of *N. limnetica* after cell damage (G. Pohnert, unpubl. data).

Interestingly, once the parasite was able to establish an infection, parasite performance was not impaired by the PUFA-rich *N. limnetica* diet. On the contrary, these hosts exhibited the highest spore load per animal. This implies that the immune system of *D. magna* is rather ineffective against *P. ramosa* once the parasite could overcome the initial defences. In general, animals reared on high PUFA food by tendency contained more spores per individual than animals reared on the moderate food source *S. obliquus*, indicating that host-parasite interactions later during the infection are subject to resource competition and that increased food quality sustains increased within-host reproduction of the parasite. Similar findings have been reported for food quantity and elemental food quality [18,44]. In accordance with previous studies [27,29], the reproductive output of healthy hosts was significantly higher on food sources containing C20 PUFAs, including supplemented diets, than on C20 PUFA-deficient food (*S. obliquus*). Similarly, infected hosts benefited from feeding on high quality algae and PUFA supplementation. The higher reproductive output of infected animals was partially due to reproduction after the parasite-induced sterile phase (castration relief). The ability to produce eggs late during the infection has been observed previously in the same combination of host and parasite clones [38]; we show here that this castration relief is clearly affected by food quality.

*P. ramosa* inherently pursues the strategy to castrate its host. Thus, resources that are normally invested in host reproduction and consequently lost to the parasite stay within the host and are available for parasite growth. Whether PUFAs or host-produced PUFA metabolites that are being retained by this re-allocation process are of special interest to the parasite cannot be conclusively stated at this point.

#### PUFA-mediated maternal effects on unchallenged and infected hosts

In the second generation experiment we found that the quality of the maternal diet has far-reaching consequences for offspring fitness with and without parasite challenge. The PUFA composition of the eggs mirrored that of the maternal food, indicating a limited capacity to modify dietary PUFAs and to adjust the allocation of specific PUFAs into the eggs. It has been reported that dietary EPA and ARA are preferentially allocated into the eggs by *D. magna*, suggesting that these PUFAs are particularly important for egg production and offspring development [43]. Even the low concentrations of ARA and EPA detected in eggs produced on the supplemented diets in our study had pronounced effects on offspring fitness. The impact of maternal PUFA supply on the reproductive output of their offspring was of unanticipated extent. Even though the offspring have never consumed PUFA-rich diets they produced the same numbers of offspring as their mothers over a period of 30 days. This is especially intriguing as the amounts of supplemented PUFAs that were allocated to a single egg were a lot smaller than the amounts the mothers received daily with their diet. Apparently, this “starter kit” provided by the mothers was sufficient to significantly improve offspring fitness. The finding that these animals managed to keep up high offspring production during 30 days suggests low C20 PUFA requirements and a strong ability to retain these PUFAs [47]. Alternatively, this could be a consequence of better developed reproductive organs in neonates maternally provisioned with PUFAs allowing for high reproductive success independent of a direct dietary C20 PUFA supply.

Under parasite challenge, effects of maternally derived PUFAs on host resistance were strikingly clear. Whenever mothers had access to dietary PUFAs the susceptibility of their offspring to infection increased more than 6-fold. It has been reported previously that mothers raised under good conditions (i.e. no stress, high food concentrations) produce offspring which are more susceptible to parasite infection [36,37,42]. A possible explanation could be that these offspring constitute a more favourable environment where resources (and especially PUFAs) are abundant and where parasites find good conditions for proliferation. Thus the situation would be similar to the one described above for the direct consumption of dietary PUFAs (resource competition). However, our results did not show increased spore production thus arguing against this possibility. This suggests that PUFA-mediated benefits for host reproduction were conveyed to the offspring in a form not accessible to the parasite. Hence, the fitness advantage linked to the maternal PUFA-supply lies primarily on the side of the host. Alternatively, animals might face a trade-off between immunity and reproduction as both are costly traits and might rely in part on the same resources [1,48,49]. Figuring that daughters of animals which have had access to dietary C20 PUFAs had already started to invest more extensively into reproduction at parasite exposure (compared to those whose mothers were raised on C20 PUFA-deficient food sources) resources might not have been sufficiently allocated towards immunity-related functions. This potential trade-off could be more pronounced in the second generation, because the offspring had no direct access to dietary PUFAs and thus relied on the limited amounts of PUFAs allocated into the eggs (in contrast to the constant supply in the first generation experiment). PUFAs can play a role in both reproduction and immunity, presumably via the action of eicosanoids, and hence we propose that the significantly higher investment in reproduction observed in offspring of mothers raised on PUFA-containing food sources has compromised defence mechanisms and thus resulted in the very high infection success.

## Conclusions

We show here that biochemical food quality can strongly affect both host and parasite fitness. Differences in resistance and reproduction can be mediated by single dietary PUFAs. Furthermore, our results pointed out that PUFA-mediated effects on the characteristics of infection are not limited to the direct consumption, but can also be conveyed to the offspring. However, direct and maternal effects may differ greatly in the extent and direction of fitness consequences for the host. Thus, food quality in general and the availability of PUFAs in particular have a great potential to affect host – parasite interactions making them a significant factor to be considered when studying disease patterns and dynamics in the field.

## Methods

### Cultivation of organisms

The experiments were conducted with a clone of *Daphnia magna* (clone HO2, originating from Hungary). Stock cultures of *D. magna* were cultivated in artificial medium (ADaM; modified after Klüttgen et al. [50]) containing 2 mg C L^-1^ of the chlorophyte *Scenedesmus obliquus* (culture collection of the University of Göttingen, Germany, SAG 276-3a).

During the life history experiment, *D. magna* were raised on either *S. obliquus*, the eustigmatophyte *Nannochloropsis limnetica* (SAG 18.99), or on the cryptophyte *Cryptomonas* sp. (SAG 26.80), which were all cultured semi-continuously in modified Woods Hole (WC) medium with vitamins [51] in aerated 5 L vessels (20°C; dilution rate: 0.2 d^-1^; illumination: 100 μmol quanta m^–2^ s^–1^). Food suspensions were produced by centrifugation of the harvested algae and resuspension in ADaM. Carbon concentrations of the food suspensions were estimated from photometric light extinctions and from previously determined carbon-extinction equations. The carbon – light extinction regressions were confirmed by subsequent carbon analysis of the food suspensions.

### Preparation of liposomes

Liposomes were prepared as described in Martin-Creuzburg et al. [29]. The amount of daily supplied ARA-containing liposomes was adjusted to provide similar carbon-based dietary ARA concentrations as in the *N. limnetica* treatment. However, we did not apply EPA concentrations as high as in the *N. limnetica* treatment, but instead provided similar amounts of ARA and EPA to be able to compare the effects potentially produced by these two PUFAs.

### Egg preparation

For the chemical analysis, second-clutch eggs of animals raised on the different food regimes were collected under a stereomicroscope by gently flushing them out of the brood chamber with a lengthened glass Pasteur pipette (Wacker and Martin-Creuzburg [43]). The eggs were washed with ultra-pure water and transferred directly into dichloromethane/methanol for subsequent fatty acid extraction (as described below). At least three *Daphnia* were used to collect a minimum of 25 eggs per sample. All eggs sampled were in the first egg stage and did not show any morphological differentiation.

### Parasite handling

For the infection of the host a clone of the Gram positive bacterium *Pasteuria ramosa* (C19, derived from a *D. magna* population from Garzerfeld, Germany and characterized in Luijckx [52] was used. Stocks of *P. ramosa* endospores were stored at −20°C within the infected host. Prior to use, the stock was thawed and the infected animal squashed in a small volume of ADaM. Endospore concentrations within these suspensions were determined under a microscope using a counting chamber (Neubauer improved).

### Life history experiments

A two generation life history experiment was conducted to assess food quality effects on healthy and *P. ramosa*-challenged *D. magna*. In the first generation experiment animals (third-clutch neonates born within 12 h) were kept individually in 80 mL of ADaM at 20°C and a 16:8 h light:dark cycle. They were randomly assigned to one of the following food regimes: *S. obliquus* (Scen), *S. obliquus* supplemented with control liposomes (+ lipo), *S. obliquus* supplemented with ARA- or EPA-containing liposomes (+ ARA, + EPA), *N. limnetica* (Nanno), or *Cryptomonas* sp. (Crypto). For the second generation experiment, mothers from the first generation were placed into fresh medium without algae shortly before the expected release of their second clutch neonates. These neonates were collected and placed individually in jars exclusively containing *S. obliquus*, irrespective of the food conditions under which they were produced. The mothers were put back into their previous food treatments. Culturing conditions corresponded to those of the first generation. All animals were transferred to fresh medium and received freshly prepared food suspensions corresponding to a total of 2 mg C L^-1^ every other day. 18 animals of each treatment were not exposed to parasite spores, 30 animals were subjected to the parasite. For infection, all animals were placed individually in 20 mL of medium at day three of the experiment and were exposed on three consecutive days to a total of ca. 12,000 *P. ramosa* spores per individual (4,000 spores per day) in the first generation experiment and to a total of ca. 6,000 spores per individual (2,000 spores per day) in the second generation experiment. This was done because of high infections rates in the first generation. Control animals in both experiments were treated as described for the spore-exposed animals; instead of infectious spores a suspension of uninfected, macerated *D. magna* was added (mock-exposure). Subsequently, animals were transferred to new, spore-free jars containing 80 mL of ADaM. Both experiments were terminated after 30 days due to expected high death rates of infected animals after approximately 40 days [53]. During this time period reproduction (viable offspring) and infection status were recorded. On day 30, all infected individuals were stored at −20°C for subsequent determination of the spore load per animal. Subsamples of infected animals of each treatment were dried for 24 h and their dry mass determined using a microbalance (Mettler Toledo XP2U; ± 0.1 μg).

### Biochemical analyses

#### Elemental composition

Aliquots of food suspensions were filtered onto precombusted glass fibre filters (Whatman GF/F, 25 mm diameter) and analyzed for particulate organic carbon (POC) and nitrogen using an EuroEA3000 elemental analyzer (HEKAtech GmbH, Wegberg, Germany). For the determination of particulate phosphorus, aliquots were collected on acid-rinsed polysulfone filters (HT-200; Pall, Ann Arbor, MI, USA) and digested with a solution of 10% potassium peroxodisulfate and 1.5 per cent sodium hydroxide for 60 min at 121°C. Soluble reactive phosphorus was determined using the molybdate-ascorbic acid method [54].

#### Fatty acids

For the analysis of fatty acids in the prepared food suspensions approximately 1 mg POC were filtered onto pre-combusted GF/F filters (Whatman, 25 mm). Total lipids were extracted three times from filters with dichloromethane/methanol (2:1, v/v). Pooled cell-free extracts were evaporated to dryness under a nitrogen stream. For the analysis of fatty acids in the liposomes, aliquots of the liposome stock solutions were evaporated to dryness directly. The lipid extracts were transesterified with 3 M methanolic HCl (60°C, 20 min). Subsequently, fatty acid methyl esters (FAMEs) were extracted three times with 2 ml of iso-hexane. The lipid-containing fraction was evaporated to dryness under nitrogen and resuspended in a volume of 20 μL iso-hexane. Lipids were analyzed by gas chromatography on a HP 6890 GC equipped with a flame ionization detector (FID) and a DB-225 (J&W Scientific, 30 m × 0.25 mm ID × 0.25 mm film) capillary column to analyse FAMEs. Details of GC configurations for the analysis of FAMEs are given elsewhere [27]. FAMEs were quantified by comparison with an internal standard (C23:0 ME) of known concentration, using multipoint standard calibration curves determined previously with lipid standards (Sigma-Aldrich). FAMEs were identified by their retention times and their mass spectra, which were recorded with a gas chromatograph-mass spectrometer (Agilent Technologies, 5975C) equipped with a fused-silica capillary column (DB-225MS, J&W). Spectra were recorded between 50 and 600 Dalton in the electron impact ionization mode. The limit for quantitation of fatty acids was 20 ng. The absolute amount of each fatty acid was related to the POC.

### Data analysis and statistics

Infection efficiencies were analyzed using a generalized linear model (GLM) with logit function as the link function for binominal distribution. Treatment effects were evaluated by assessing deviation from the grand mean. Numbers of offspring produced on the different food regimes were analyzed using a GLM with log function as the link function for quasi-Poisson distribution. To compensate for overdispersion the model was fitted using quasi-Poisson errors [55]. To specify differences among food regimes the subsets “control” and “infected” were analyzed separately. For both GLMs, multiple comparisons among food regimes were conducted with the ‘multcomp package’ in R (R Development Core Team, 2010) using general linear hypotheses testing as an implementation of the framework for simultaneous inference according to Hothorn et al. [56]. To test for differences in within-host reproduction of the parasite between food treatments one-way analyses of variance (ANOVA) were carried out followed by multiple comparisons (Tukey’s HSD); assumptions for ANOVA were met. All analyses were performed using the statistical software package R (v.2.12.0).

## Competing interests

The author(s) declare that they have no competing interests.

## Authors’ contributions

NS and DMC planned the experiment and wrote the manuscript. NS carried out the experiments and analysed the data. DE contributed to the planning of the study, to the interpretation of the results and to revising the manuscript. All authors approved the publication of the study.
